# Leveraging existing education innovations to establish a community of practice to promote medical education scholar development

**DOI:** 10.1080/10872981.2022.2133587

**Published:** 2022-10-11

**Authors:** R. Logan Jones, Amy Miller Juve, Reem Hasan, Alexandra Shuford, Patricia A. Carney

**Affiliations:** a Department of Internal Medicine, Oregon Health & Science University, USA; b Department of Anesthesiology and Perioperative Medicine, Oregon Health & Science University, USA; c Departments of Internal Medicine and Pediatrics, Oregon Health & Science University, USA; d Undergraduate Medical Education Assessments, Oregon Health & Science University, USA; e School of Medicine, Oregon Health & Science University, USA

**Keywords:** Community of practice, health professions education scholarship, faculty development, learner engagement

## Abstract

**Problem:**

While some academic health centers have organizational structures to support educational scholarship, such as Medical Education Research Units (MERU), many education scholars may lack access to such institutional resources to support their research agenda and professional growth.

**Approach:**

In 2014, as part of an externally funded education grant, three faculty educators established a unique education data management system Research & Evaluation Data for Educational Improvement (REDEI). Lacking an institutional MERU, they established an educational research community of practice (CoP) using REDEI as the research dataset. The senior faculty member’s effort to facilitate the group is funded by the Dean’s Office.

**Outcomes:**

The CoP meets every 2 weeks to generate research ideas, discuss analytic approach and strategy, review analyses designed to address or explore a research question, and plans for manuscript development. Our CoP has grown from 3 to 18 members representing faculty educators, administrators, and staff across many departments in the School of Medicine. As of 2021, the REDEI system contains performance data on 1,246 students across all years of undergraduate medical education. To date, we have published 11 peer-reviewed educational research manuscripts. Five learners have served as coauthors (three medical students and two residents), three of whom were first authors. Eleven additional papers are in process. This community of practice supports productivity, provides mentorship, overcomes barriers, and is flexible enough for people to join when they can or when an area of interest is actively under development.

**Next Steps:**

We are working on educational interdisciplinary research grant submission and creating collaborations with other institutions. Our focus remains on honing skills in grantsmanship, identification of impactful research questions, application of rigorous methods and instrumentation to address them, and refining process of budget development, timelines, and other planning strategies.

## The problem

Participating in the scholarship of medical education enables educators to learn from each other, build on their achievements, and career advancement. While many medical education professionals derive deep and intrinsic rewards from their scholarly work, those who must also publish for promotion and tenure may also face external pressures to pursue a high level of scholastic output without the support that researchers in clinical medicine or basic sciences often have. Regardless of motivation for performing medical education scholarship, scholars in medical education face a myriad of obstacles to successfully advance educational research. These include, but are not limited to: a lack of training in educational research methods, design, and statistical analyses; lack of appreciation for educational rigor and scholarship; limited opportunities for extramural funding; time demands due to competing responsibilities and lack of protected time; and a need for mentorship, community, accountability, and rewards [1–3].

Varying organizational structures have been developed to support the academic mission of educational researchers, such as the creation of formal offices or departments of medical education or medical education research units (MERU) [4]. In the USA, only 34% of US-based medical schools having an active medical MERU as recognized by the Society of Directors of Research in Medical Education [5]. Another contemporary model called Health Professions Education Scholarship Units (HPESU) has emerged as a less formal structure relative to offices or departments of medical education. HPESUs are a discrete organizational structure sponsored by a parent organization and typically are constituted primarily with three professional HPES roles: (1) clinician educators, (2) research scientists, and (3) administrative leaders [6,7] who work together to produce health professions education scholarship. While MERUs and HPESUs offer for support for scholarship, what should education scholars do if their parent organization lacks this organized framework?

Originally proposed in 1991 by Lave and Wenger, communities of practice (CoP) represents a conceptual framework that describes a group of ‘people who share a concern or a passion for something they do and learn how to do it better as they interact regularly’ [8]. Over time, a CoP builds collective growth among members through mutual engagement, joint enterprise, and shared repertoire [9]. Thus, a CoP aims to foster learning and growth via peer mentorship and shared practice among a group of people who share a common passion [9]. This is contrasted against a typical workgroup or committee, which typically focus on productivity as a chief outcome. Early experiences with CoPs formed among medical educators show promise for breaking down silos and bringing medical educators from various backgrounds together [10,11]. Moreover, purposeful cultivation of CoPs have been proposed as a model to address many lacking aspects of traditional educator development efforts [12,13]. At Oregon Health & Science University (OHSU), there is no MERU nor a formally recognized HPESU. However, building off of previous education innovation work, namely, receipt of an American Medical Association Accelerating Change in Medical Education Grant, we created an educator’s CoP to foster educator development toward supporting research and scholarship skills. In this innovation report, we describe how we formed a CoP, its associated costs, benefits to its members, as well as scholarly outcomes.

## Approach

We received funding to transform OHSU’s undergraduate medical education (UME) curriculum from a time-based to time-variable competency-based program from the AMA [14]. The support included the development of a relational data system with an Internet-based learner portfolio viewing portal designed to allow students, faculty educators, advisors, and educational leaders to monitor the progression of student development in as close to real time as possible. Fed by multiple data streams, such as ExamSoft, MedHub, Anatomy Hive and USMLE/NBME, the **R**esearch & **E**valuation **D**ata for **E**ducational **I**mprovement (REDEI) system houses student performance on a variety of formative and summative educational metrics. REDEI’s dashboards include performance on weekly quizzes, end of block examinations (e.g., OHSU final exams, clinical skills and performance exams, USMLE/NBME assessments, and health systems science exams) [15], as well as workplace-based assessments (WBAs) aligned with the 13 Core Entrustable Professional Activities (EPAs) for entering residency [16]. Scholarly projects, student artifacts, preceptorship, and remediation data are also included in REDEI.

When REDEI was initially developed, we applied for and received Institutional Review Board (IRB) and Family Education Rights and Privacy Act (FERPA) Approvals for use of REDEI Data for research purposes [17] and additionally received a Federal Certificate of Confidentiality to protect the privacy of research subjects by prohibiting disclosure of identifiable, sensitive research information to anyone not connected to the research except in a few specific situations [18]. Now well established, the REDEI system refreshes every two hours in its data processing and viewing systems.

Development of the REDEI system involved a senior faculty member and educational researcher (Author PAC) team leader, a web developer, and an assistant dean for student affairs. This team, known as the REDEI team, met every other week beginning in 2014 to develop, review, and revise REDEI features as ongoing needs were identified. By 2016, the REDEI Data system had captured performance data on 453 medical students across a variety of measures, and data were stable enough to be used for educational research. As the structural and operational features of the portfolio system matured (Table 1), the REDEI team began to grow and was replaced with the **R**EDEI User **S**cholarship **H**opefuls (RUSH) team.

**Table 1.** Evolution of the REDEI/RUSH Learning Community Team.

Time Period	Learning Community Team Members	Educational Research Focus
2014–2015	*REDEI Team (n = 3)* Educational researcher – team lead Web designer/programmer Assistant dean for student affairs	Develop data structures and interfaces between data systems Develop viewing portal prototypes Revise data structures, data systems and viewing portal as needed.
2016–2020	*REDEI Team (n = 9)* Educational researcher – team lead Web designer/programmer Assistant dean for student affairs Assistant dean for special projects Education faculty from neurology, infectious disease, anesthesiology Program manager for UME assessments Director of UME assessments	Assessed data quality and created corrective systems for automated data hygiene Began developing and tracking educational research ideas using a tracking database Developed and implemented supplemental data collection as needed based on research ideas Revised IRB as needed Conducted analyses and developed and submitted manuscripts for peer review
2020–Present	*RUSH Team (n = 18)* Educational researcher – team lead Web designer/programmer Assistant dean for student affairs Assistant dean for special projects Education faculty from neurology, infectious disease, anesthesia, pulmonary critical care, internal medicine (n = 5), 2 fellows Program manager for UME assessments Director of UME assessments	Developed and implemented a workplace-based assessment app that collected data using hand-held devices (e.g., smartphones, tablet computers) Data warehouse expands Educational research tracking database expands Research projects and analyses are prioritized.

The RUSH team made this transformation to address the institution’s desire to develop educators interested in learning educational research and academic writing skills. Accordingly, we intentionally developed RUSH as an educator’s CoP using the definition espoused by McGrath *et al*., as discussed in “The Problem” section [9]. With the data supplied by the REDEI system as the convening platform, the RUSH team founders sought to build a community in which members’ skill development was the primary objective and scholastic output regarded as a secondary outcome.

### Operational features of our community of practice

CoPs aim to foster learning and growth via peer mentorship and shared practice among a group of people who share a common passion [9]. To create a CoP, the RUSH team developed and came to consensus on a shared goal. Namely, that we would be dedicated to helping all team members’ skills to rise together toward (1) identifying relevant educational research questions; (2) providing input into preliminary and final analyses; and (3) ongoing manuscript development while engaging and including learners and junior faculty whenever possible. Given our CoP’s roots, our shared task is to research questions arising from or in relation to the evolving REDEI dataset.

The initial core REDEI group of 3 grew to 9 members in 2016, to now 18 core members of the RUSH team who attend bi-weekly meetings when possible (Table 1). RUSH has amassed a broad array of educators from various departments and of differing ranks who would not otherwise collaborate in educational research, including medical student and resident members who are working on scholarly projects related to their training. Membership has been variable but steady with some standing members, while others have come and gone depending on their interest areas and the CoP focus.

RUSH team meetings begin with a *Research Jeopardy* game where participants choose correct findings among incorrect ones from recently published educational research projects. The game is designed to help members recognize the often-wrong assumptions commonly made about educational research outcomes and to support critical thinking and assessment of research rigor. The remainder of the time is typically spent identifying and discussing potential projects, reviewing data/making decisions on data quality (e.g., inclusion/exclusion criteria, management of outliers), discussing the results of analyzed data, and identifying and planning next steps for manuscript development. We use a research idea/manuscript tracking system to manage progress. Participants sign up in the tracking system to work on a project of interest, which includes manuscript title, lead and senior authors, writing group, date of data maturity, target journal, and submission timeline. The tracking database provides the entire group with a shared mental model from which to work, starting with the research idea and following through to manuscript development phases, submission status, and revising/resubmission management. The tracking database helps the CoP maintain a shared vision.

We use the International Committee of Medical Journal Editors’ (ICMJE) four-point authorship criteria to guide author responsibilities, where all authors must meet *all* four criteria: (1) substantial contributions to the conception or design of the work; or the acquisition, analysis, or interpretation of data for the work; (2) drafting the work or revising it critically for important intellectual content; (3) final approval of the version to be published; and (4) agreement to be accountable for all aspects of the work in ensuring that questions related to the accuracy or integrity of any part of the work are appropriately investigated and resolved [19].

After projects and team members are identified, and next steps delineated, team members work on manuscript development using a two-group approach: Group 1 produces the first rough draft while Group 2 undertakes critical revisions. We have found it helpful to save fresh eyes for Group 2 work, as it serves as a ‘mini review.’ We aim for no more than four drafts prior to submission, and the objective for each review is clearly conveyed prior to work on the respective draft. We have also found that everyone gains valuable experience by taking turns on each writing group, according to their interest and available bandwidth during the production of the manuscript. If someone signs up for an authorship role but is unable to fulfill the four responsibilities, the lead and senior authors remind them of the criteria and ask if they feel they have met them. Thus far, this approach has not changed the dynamic of the CoP, and the authors who have not met criteria have either stepped up to meet them or have agreed to be removed from the paper.

### Institutional support and sustainability

The educational researcher team lead, a senior faculty member who devotes 0.20 FTE to the effort, is funded by the Dean’s Office. This a funding mechanism which bypasses more traditional departmental or office funding models. This individual (1) manages the agenda, develops Research Jeopardy questions, and facilitates RUSH meetings; (2) oversees and conducts data hygiene activities; (3) conducts data analyses; (4) develops the research methods and results sections of manuscripts; (5) provides mentoring on manuscript development and publishing ethics; and (6) assists with grant development and submissions to advance the educational research effort. The Web-designer/programmer who works on data capture and structures and creates analytic files dedicates 1.0 FTE.

## Outcomes

### Benefits of the RUSH Community of practice

RUSH participants receive mentoring in educational research that is often not available in their departments due to a lack of expertise or competing demands. As part of this mentoring, their skills in manuscript development, revision, and resubmission improve and they make meaningful progress in their scholastic mission. Similarly, they receive assistance with educational grant development, submission, and, if funded, project management that further advances their skills as educational researchers. The collaborative ideas and newly formed partnerships across departments and divisions have been instrumental in contributing to a highly functional CoP.

Since 2016, members of our CoP have published 11 peer-reviewed educational research manuscripts, with an additional one currently in review (Table 2). Five learners have served as coauthors on these papers (three medical students and two residents), three as first authors. We began publishing with REDEI data in 2017, as it took about three years for the REDEI system to be mature enough to use data for research purposes. We are able to produce one to two papers each year. We have an additional 11 papers in process between the stages of conceptualization and statistical analyses. Table 3 provides additional metrics from the RUSH group, including conference proceedings, program evaluations, quality improvement/curricular revision, and grant submissions and receipts. We have succeeded in establishing a growing community of scholars who fit this work into their schedules because it fosters and supports productivity, helps to overcome barriers by working together so everyone’s skills improve, and is flexible enough for people to join when they can or when an area of interest is actively under development.

**Table 2.** Summary of Published Sholarship Arising From RUSH CoP.

Published Papers	Focus of the Work and Pertinent Findings
Thayer EK, Rathkey D, Miller MF. Applying the institutional review board data repository approach to manage ethical considerations in evaluating and studying medical education. *Med Educ Online*. 2016;21:10.3402/meo.v21.32021. doi:10.3402/meo.v21.32021	Addressed IRB issues related to research in medical education, and proposed a novel IRB repository approach being used by both of the medical schools profiled in the paper.
Deiorio NM, Carney PA, Kahl LE, Bonura EM, Juve AM. Coaching: a new model for academic and career achievement. *Med Educ Online*. 2016;21:10.3402/meo.v21.33480. doi:10.3402/meo.v21.33480	Identified constructs and relevant definitions for academic coaching in medical education based on coaching literature, which focus on (1) relationship principles, (2) assessing learners, (3) creating action plans, and (4) assessing and revising plans as needed.
Carney PA, Haedinger LA, Kahl LE, Deiorio NM, Bonura EM, Kraakevik JA. the association between assigned independent learning schedule and medical student performance on examinations. *Medical Science Educator*. 2017;27(2):253–257. doi:10.1007/s40670-017-0389-1	Tested the hypothesis that students assigned to independent learning on Thursday afternoon would perform better on Friday quizzes compared to students assigned independent learning time on Monday or Tuesday. No statistical differences were noted.
Yeager L^a^, Valenzuela S, Marino M, Carney PA. An observational study of the impact of attendance on pre-clinical undergraduate medical education performance. *Educational Research Applications*. Published online 28 February 2018. Accessed 17 January 2022. https://www.gavinpublishers.com/articles/research article/educational-research-applications-issn-2575-7032/an-observational-study-of-the-impact-of-attendance-on-preclinical-undergraduate-medical-education-performance	Tested the hypothesis that attendance during basic science lectures and labs affects performance on end of block examinations. Attendance at ≥80% was not consistently associated with improved examination scores.
Carney PA, Mejicano GC, Bumsted T, Quirk M. Assessing learning in the adaptive curriculum. *Med Teach*. 2018;40(8):813–819. doi:10.1080/0142159X.2018.1484083	Two medical school faculty teams describe how they have approached assessments conducted in adaptive curriculum, including using portfolio systems to improve learning and curricula continuous improvement cycles.
Najibi S^a^, Carney PA, Thayer EK, Deiorio NM. Differences in coaching needs among underrepresented minority medical students. *Fam Med*. 2019;51(6):516–522. doi:10.22454/FamMed.2019.100305	Exploratory study that investigated student perceptions and coaching needs among URM and non-URM medical students Racially, ethnically, and socially underrepresented minority students have different coaching needs compared to majority students which coaches do not recognize. Faculty and program development is warranted.
Carney PA, Bonura EM, Kraakevik JA, Juve AM, Kahl LE, Deiorio NM. Measuring coaching in undergraduate medical education: the development and psychometric validation of new instruments. *J Gen Intern Med*. 2019;34(5):677–683. doi:10.1007/s11606-019-04888-w	This study reports on the development and psychometric assessments of two instruments, one to assess medical students’ perspectives of coaching and perspectives of coaching by coaches. The result is robust measurements for this educational paradigm.
McKerrow I^a^, Carney PA, Caretta-Weyer H, Furnari M, Miller Juve A. Trends in medical students’ stress, physical, and emotional health throughout training. *Med Educ Online*. 2020;25(1):1,709,278. doi:10.1080/10,872,981.2019.1709278	This study used the SF-8 and the Perceived Stress Scale at baseline (matriculation) and at the end of Years 1, 2, and 3 to identify when during medical school students ‘wellness is poorest, which occurred at the end of Year 1, but improved thereafter, while perceived stress remained high and unchanged.
Kraakevik JA, Frederick M^a^, Ryan N, Haedinger LA, Carney PA. An observational study of an approach to accommodate a fourth-year to third-year neurology clerkship curricular transition. *Med Educ Online*. 2020;25(1):1,710,331. doi:10.1080/10,872,981.2019.1710331	This paper reports on learner outcomes in a fourth-year neurology clerkship when, to address a bulge in medical student placements associated with a curricular transformation, students could opt out of taking the neurology clerkship if they undertook a complete neurological history and physical exam that was observed by a neurology faculty member. No differences were found between study groups on the US-NBME clinical neurology subject exam.
Kraakevik JA, Beck Dallaghan GL, Byerley JS Managing expansions in medical students’ clinical placements caused by curricular transformation: perspectives from four medical schools. *Med Educ Online*. 2021;26(1):1,857,322. doi:10.1080/10,872,981.2020.1857322	Four different medical school faculty team describe how they addressed the impact of curricular transformation on clerkships using an implementation science lens. Four different approaches were used to managing the ‘bulge’ as classes overlap in clerkships, which are described.
Hasan R, Phillipi CA, Smeraglio A, Blank J^a^, Shuford A, Budd C, Garcia A, Carney PA. Implementing a real-time workplace-based assessment data collection … MedEdPublish | Open Access Publishing Platform. doi:10.15694/mep.2021.000022.1	A novel WBA system designed for handheld devices that load data to a repository using the internet for EPA competency assessment. Is described along with initial results from our inaugural medical student cohort.

^a^Denotes author was a student or resident at time of work.

**Table 3.** Other RUSH Activities.

Other RUSH Activities	*n*
Conference proceedings	6
Program evaluations	11
Quality improvement/Curricular revision projects	Ongoing
Grant submissions	4
Grants received	2

## Next steps

As the sophistication of our CoP has grown, we have begun collaborating on educational research grant submissions, many of which are multi-institutional and interdisciplinary. This provides additional skills in grantsmanship, including networking and relationship development, to advance educational research beyond a single setting. In addition to fostering advancement of junior faculty, success in grant attainment provides protected time and other resources to conduct studies that are currently added on to busy schedules. To conduct more impactful research than we are currently able to do at our own institution, we have also requested datasets from the AAMC to conduct larger studies, such as identifying predictors of success among students who are underrepresented in medicine. Finally, we are exploring how to leverage our CoP process and shared expertise to support projects outside of our original focus of REDEI platform research questions. We have found that the CoP approach has fostered educational scholarship effectively at our institution and hope that other academic institutions in medicine may benefit from the community of practice model we have implemented, as it may be generalizable to other settings. The Health Professions Education Scholarship Units (HPESU), another possibility, would require more institutional support than is currently available at OHSU.
